# Fibula Nail versus Locking Plate Fixation—A Biomechanical Study

**DOI:** 10.3390/jcm12020698

**Published:** 2023-01-16

**Authors:** Felix Christian Kohler, Philipp Schenk, Theresa Nies, Jakob Hallbauer, Gunther Olaf Hofmann, Uta Biedermann, Heike Kielstein, Britt Wildemann, Roland Ramm, Bernhard Wilhelm Ullrich

**Affiliations:** 1 Department of Trauma, Hand and Reconstructive Surgery, Jena University Hospital, Friedrich Schiller University Jena, 07747 Jena, Germany; 2 Department of Science, Research and Education, BG Klinikum Bergmannstrost Halle gGmbH, 06112 Halle, Germany; 3 Department of Trauma and Reconstructive Surgery, BG Klinikum Bergmannstrost Halle gGmbH, 06112 Halle, Germany; 4 Institute of Anatomy I, Jena University Hospital, Friedrich Schiller University Jena, 07743 Jena, Germany; 5 Institute of Anatomy and Cell-Biology, Halle University-Hospital, Martin Luther University, 06108 Halle, Germany; 6 Fraunhofer Institute for Applied Optics and Precision Engineering (IOF), Albert-Einstein-Str. 7, 07745 Jena, Germany

**Keywords:** trauma surgery, open reduction and internal fixation, syndesmosis, upper ankle joint, syndesmotic screw, biomechanical, osteosynthesis, geriatric fracture, geriatric trauma, fibular nail

## Abstract

In the treatment of ankle fractures, complications such as wound healing problems following open reduction and internal fixation are a major problem. An innovative alternative to this procedure offers a more minimally invasive nail stabilization. The purpose of this biomechanical study was to clarify whether this method was biomechanically comparable to the established method. First, the stability (range of motion, diastasis) and rotational stiffness of the native upper ankle were evaluated in eight pairs of native geriatric specimens. Subsequently, an unstable ankle fracture was created and fixed with a locking plate or a nail in a pairwise manner. The ankles showed significantly less stability and rotational stiffness properties after nail and plate fixations than the corresponding native ankles (*p* < 0.001 for all parameters). When comparing the two methods, both showed no differences in their range of motion (*p* = 0.694) and diastasis (*p* = 0.166). The nail also presented significantly greater rotational stiffness compared to the plate (*p* = 0.001). However, both fixations remained behind the native stability and rotational stiffness. Due to the comparable biomechanical properties of the nail and plate fixations, an early weight-bearing following nail fixation should be assessed on a case-by-case basis considering the severity of fractures.

## 1. Introduction

Ankle fractures are among the most common fractures experienced by individuals [1,2,3]. Dislocated ankle fractures and injuries involving the syndesmotic complex are usually treated surgically to restore the integrity of the ankle joint [4]. Open reduction and internal fixation (ORIF) using plate and screws is an established standard practiced in the field [5,6,7].

Older age and comorbidities lead to higher rates of complications ranging from 7 to 13% as a result of ORIF [8,9,10,11,12]. 

Closed reduction and nail fixation (CRNF) is an alternative minimally invasive treatment option for ankle fractures [13,14,15,16,17]. Less frequent complication rates [18,19,20,21] and the immediate possibility of full weightbearing postoperatively [18,22] seem to be the advantages of CRNF.

To the authors’ knowledge, to date, there are only three relevant biomechanical studies on human specimens on the fibula nail [10,11,23]. 

Smith et al. compared intramedullary fibular nail fixation using one fibulotibial syndesmotic screw with non-locking plate fixation without a fibulotibial syndesmotic screw in an OTA/AO B-Typ fracture model. They observed greater torque to failure and a better preservation of the fibular construct in the nail group [10]. The main limitation of this study seems to be that only in the nail group was a fibulotibial syndesmotic screw used. However, a fibulotibial syndesmotic screw was not necessary for the fracture simulated in this study with plate osteosynthesis.

Switaj et al. compared the nail with a locking plate using only one syndesmotic screw in each group. They observed the nail to present less external rotational stiffness in highly unstable ankle fractures, while syndesmotic diastasis exhibited failure characteristics comparable to a locking plate [11]. One limitation of this study is that only one fibulotibial syndesmotic screw was used, providing the nail with less potential for rotational stabilization.

Carter et al. created an OTA/AO B-Type fracture and tested fibula nail fixation with one fibulotibial syndesmotic screw against a locking plate fixation without a fibulotibial syndesmotic screw. They observed no significant differences when testing to failure [23]. The biomechanical results remained inconsistent regarding the achieved biomechanical stability.

The present research investigated the biomechanics of fibula nail fixation using both fibulotibial syndesmotic screws and compared it with a locking plate fixation using two fibulotibial syndesmotic screws. A fracture model corresponding to a highly unstable injury was attempted to simulate a worst-case scenario. We aimed to address the above-mentioned limitations of the pre-studies.

We hypothesize that the fixation of rotationally unstable ankle fractures with a fibula nail is biomechanically comparable to fixation using a locking plate regarding the stabilization of the syndesmotic complex and rotational stiffness. Furthermore, the stability of both fixation methods was compared to the native, non-fractured condition.

## 2. Materials and Methods

### 2.1. Specimen

For the present study, eight fresh, deep-frozen (≤−20 °C) lower-leg specimens following disarticulation in the knee joint were used (six males and two females, age: 86 ± 6 years). The donors’ history did not include musculoskeletal diseases or known injuries of the upper ankle joint. To analyze the bone quality and exclude relevant differences between the specimens, each frozen specimen underwent diagnostic computed tomography (CT) and bone mineral density (BMD) measurements by quantitative computed tomography (qCT) in the cancellous metaphyseal regions of the tibia, the distal fibula, and the talus body (Device GE Revolution EVO, 128 lines, Solingen, Germany).

**Figure 1:**
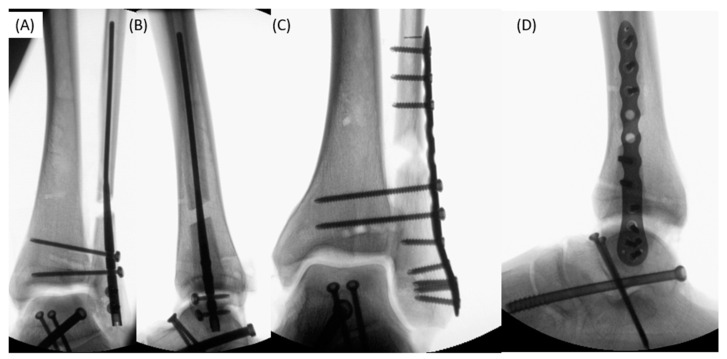
X-ray (**A**,**B**) fixations with the Vitus-Fi Fibula Nail System (nail group) with two locking and two syndesmotic screws in anterior–posterior and lateral radiographs; X-ray (**C**,**D**) fixations with locking plate (plate group) with two syndesmotic screws in the anterior–posterior and lateral radiographs.

For further biomechanical testing, the specimens were thawed over 18 h. Initially, the muscles and soft tissue up to 10 cm above the joint level of the upper ankle were removed. In order to be able to detect only the movements of the upper ankle joint at the syndesmotic level, arthrodesis of the lower ankle joint and Lisfranc joint was performed using talocalcaneal screws (Fa. Synthes, Johnson & Johnson Medical GmbH, Norderstedt, Germany, diameter: 4 mm, length: 60 mm, Figure 1). In addition, the hindfoot was fixed with a spongiosa screw (Fa. Stryker, diameter: 6 mm, length: 85 mm, Figure 1). The foot was submerged in methylmethacrylate (PMMA, TECHNOVIT^®^, Kulzer GmbH, Hanau, Germany) and the tibial plateau was fixed into portable frames using Schanz screws.

### 2.2. Standardized Instability and Fixation

**Figure 2:**
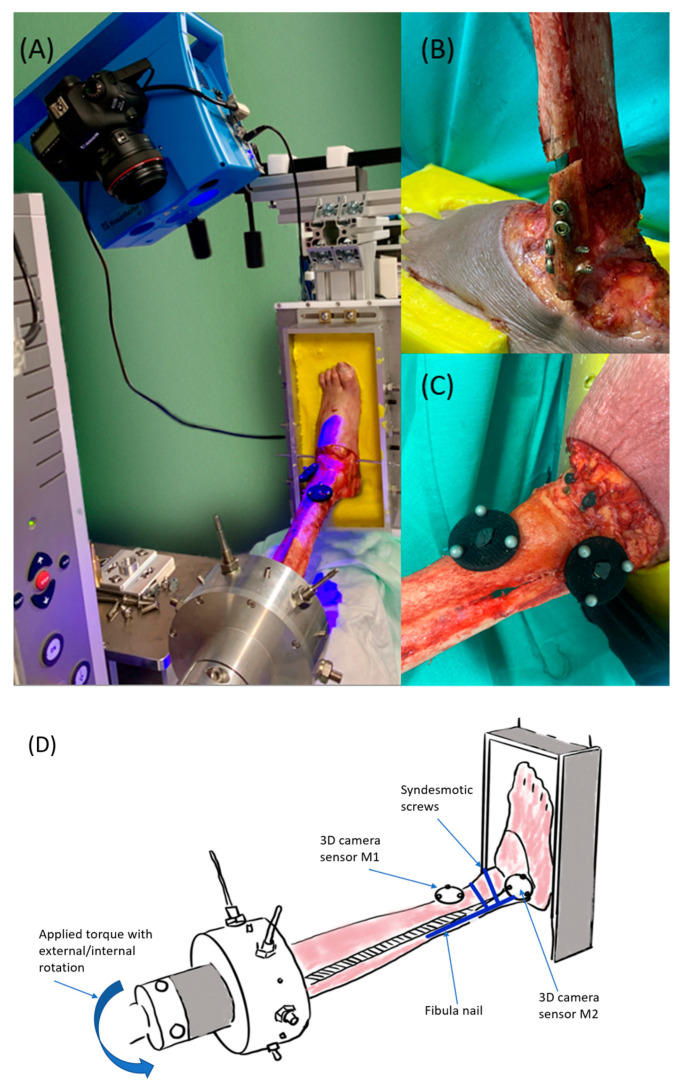
(**A**) The test setup with the material testing machine and optical 3D measuring system, (**B**) a prepared specimen with implanted vitus fibula nail, (**C**) the arrangement of the marker plates M1 (distal tibia) and M2 (distal fibula), (**D**) test setup with the placement of the marker plates M1 and M2. Torque was applied by repeated external/internal rotations of the tibia plateau against the fixed foot.

A standardized lesion simulating a Weber C fracture with rotationally unstable pronation external rotation (PER) injury according to Lauge–Hansen (stage 4) was set (Figure 1C and Figure 2B). For this purpose, an osteotomy of the fibula was performed at a 45° angle to the axis using an oscillating saw 1.5 cm above the syndesmotic level. To generate the relevant instability within the fracture zone, a 0.5–1 cm wide bone segment was removed at this height to simulate a zone of fragmentation. The entire syndesmotic complex (anterior, intermedius, and posterior talofibular ligaments) and the deltoid ligament were cut. The interosseous membrane was distally incised up to the fracture level. All other ligaments or bones remained intact.

For the fixation, the donors’ left and right specimens were randomized in plate or nail groups to avoid side-specific bias.

The following fixation methods were used:

Nail group: The Vitus-Fi Fibula Nailing System (Fa. Dieter Marquardt Medizintechnik GmbH, Spaichingen, Germany) was used following the surgical instructions. Distally, the nail was locked twice with screws and two tricortical syndesmotic screws were placed over the target system (Figure 1A,B)

Plate group: A locking plate fixation (Variax, Fa. Stryker, Duisburg, Germany, 12-hole plate) was performed. Two fibulotibial tricortical syndesmotic screws were inserted in addition to the locking screws (Figure 1C,D).

Fixation was performed by two of the authors (FK and BU) who were surgically experienced to avoid creating differences in the quality of the fixations performed.

### 2.3. Experimental Setup

**Figure 3:**
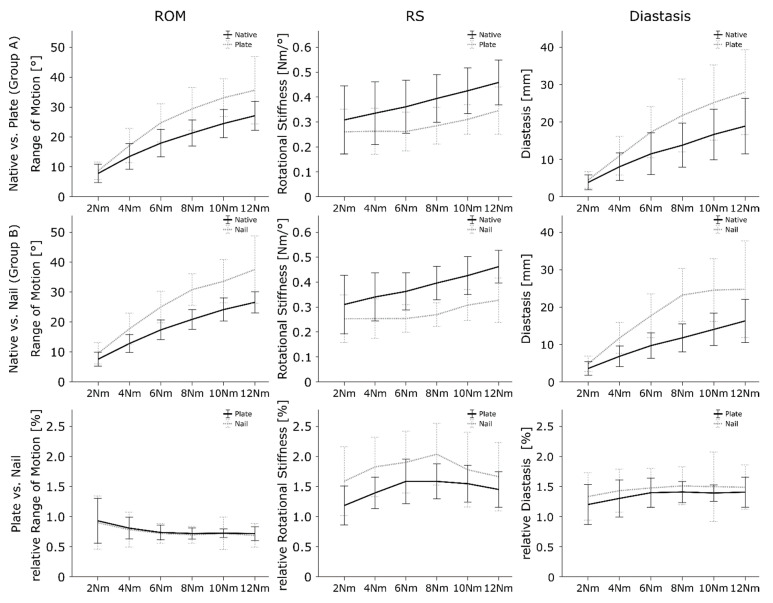
Results for the ROM, RS, and diastasis for native condition versus plate fixation (row 1), native condition versus nail fixation (row 2), and plate versus nail fixation (row 3). The data are presented with 95% confidence intervals. Non-overlapping intervals indicate significant differences.

The biomechanical tests were performed with a constant axial preload of 750 N by a pneumatic device (to simulate body weight). A material testing machine (zwickiLine Z1.0 from Zwick/Roell, Ulm, Germany) was used to expose standardized rotational torque to the specimens (Figure 2A). The actuator of the testing machine was connected by a lever arm to the portable frame in which the specimen was proximally fixed. Therefore, the external and internal rotations of the tibial plateau could be applied against the fixed foot, simulating the external or internal rotations of the foot (Figure 2A and Figure 3). Rotational loads were applied starting at 2 nm and were subsequently increased in 2 nm steps up to 12 nm. A total of 10 cycles in each direction (external and internal rotations) were performed. First, the loading cycles were performed on the native, followed by the testing of the destabilized and osteosynthesized specimens.

#### 2.3.1. Movement Measuring

During the biomechanical tests, the movements of the distal fibula and tibia were recorded using an optical 3D measurement system that tracks two marker plates (kolibri CORDLESS, Fraunhofer IOF, Jena, Germany; measurement uncertainty of 20–100 µm) [24]. Each marker plate consisted of three passive, spherical markers 6.5 mm in diameter attached to a jet-black plate with a 2.5 cm diameter. The marker plates (hereafter referred to as M1 and M2) positioned tibially (M1) and fibularly (M2) were applied in the same manner to the syndesmotic plane on each specimen, as shown in Figure 2C and Figure 3.

#### 2.3.2. Biomechanical Parameters

In order to compare the stability of the native and stabilized specimens, and both fixation methods, the following parameters were used: the angle of rotation between the fibula and tibia (ROM), rotational stiffness (RS), and the diastasis between the fibula and tibia. These parameters were measured and calculated as follows.

#### 2.3.3. Range of Motion (ROM)

The ROM in degree was measured as the transversal angle of rotation in the tibial plateau against the fixed embedded foot. The ROM was recorded for each load level as the sum of the maximal degree of the internal and external rotational angles. The greater the ROM, the greater the movement in the ankle joint and thus the instability.

#### 2.3.4. Rotational Stiffness (RS)

For each load level, the RS in Nm/° was calculated based on the applied force in Nm and the angle of rotation of the tibia against the foot in the transversal plane. The data were calculated separately for external and internal rotations. The higher the value of RS, the greater the rotational stiffness, and consequently the stability of the upper ankle joint.

#### 2.3.5. Diastasis

To evaluate the diastasis in mm, the maximum change in distance between M1 (tibia) and M2 (fibula) at each load level was measured.

#### 2.3.6. Normalized ROM, RS, and Diastasis

To compare the stability between both fixation methods, avoiding side-related bias, ROM, RS, and diastasis were normalized by the results in the native condition for each load level, respectively.

### 2.4. Statistics

The differences in the mean BMD between the plate and nail groups were analyzed using Welch tests. A statistical analysis of the differences between native and stabilized specimens (fixed factor) was performed using separate general linear mixed models (GLMs) using ROM, RS, and diastasis as dependent variables. Bonferroni post hoc tests were used to perform pairwise comparisons. To compare both stabilization methods, the normalized values of ROM, RS, and diastasis were used in separate GLMs with the group as the fixed factor, respectively.

Due to the small sample size, effect sizes (ESs) such as Cohen’s d (0.2 = small, 0.5 = medium, 0.8 = large effects) were presented for the comparison of fixation versus native condition and the comparison of both methods for the main effects, in addition to *p*-values. If a significant interaction effect between the load level and fixed factor could be observed, the *p*-values were presented. Otherwise, no statement was exhibited. Descriptive statistics are presented as the mean and standard deviation. For a visual comparison and statistical interpretation of the pairwise comparisons, the results were presented graphically as the means and 95% confidence intervals as error bars. This means that the true mean is within these limits 95% of the time and non-overlapping error bars indicate significant differences.

SPSS version 26 (IBM SPSS Statistics for Windows, IBM Corp., Armonk, NY, USA) was used for statistical analyses. The significance level was set to *p* = 0.05.

## 3. Results

### 3.1. Bone Mineral Density

The BMD did not differ between both groups (*p* = 0.943), with 226 ± 62 g/cm^3^ in the plate group and 229 ± 59 g/cm^3^ in the nail group (*p* = 0.943). Thus, the BMD was assumed to not be different between the two sides, and the effect of the BMD on the stability of the fixation was assumed to produce an effect in a comparable manner.

### 3.2. Native versus Plate and Nail

#### 3.2.1. Range of Motion

When comparing the ROM of the specimens in the plate group with the corresponding native specimen, the fixed specimens showed significantly greater ROM values (*p* < 0.001; native: 18.7 ± 8.0°; plate: 24.8 ± 11.0°; ES: 0.51, Figure 3). Furthermore, the ROM was significantly affected by the torque level; as the torque level increased, the ROM also increased (*p* < 0.001).

The ROM of the specimens following fibula nail fixation significantly differed from the native specimens (*p* < 0.001; native: 18.2 ± 7.5°; nail: 25.7 ± 11.0°; ES: 0.65, Figure 3). The ROM was again significantly affected by the level of torque (*p* < 0.001).

#### 3.2.2. Rotational Stiffness

The stiffness significantly decreased after plate fixation compared to the native situation (*p* < 0.001; native: 0.38 ± 0.12 Nm/°; plate: 0.29 ± 0.08 Nm/°; ES: 1.39, Figure 3). The upper ankle joints of the specimen with the fibula nail fixation showed significantly less RS compared to the native specimens (*p* < 0.001; native: 0.38 ± 0.11 Nm/°; nail: 0.28 ± 0.08 Nm/°; ES: 1.15, Figure 3). For both techniques, the load level showed no significance in the post hoc pairwise comparisons (*p* < 0.085).

#### 3.2.3. Diastasis

The diastases were significantly greater after plate fixation compared to the native condition (*p* < 0.001; native: 12.1 ± 7.6 mm; plate: 18.0 ± 10.7 mm; ES: 0.83, Figure 3). Diastasis increased with greater loads (*p* < 0.001).

A significant greater diastasis value was observed for the nail fixed specimen also comparted to native tissue (*p* < 0.001; native: 10.4 ± 5.9 mm; nail: 17.7 ± 9.7 mm; ES: 0.83) (Figure 3). With an increase in the load level, the diastasis also increased (*p* < 0.001). A significant interaction effect between nail fixation and load level was observed with *p* = 0.010.

### 3.3. Plate vs. Nail

#### 3.3.1. Range of Motion

Comparing the two methods, no significant difference and low ES in the normalized ROM was observed for the mean of all force levels (*p* = 0.694; plate: 0.77 ± 0.22; nail: 0.75 ± 0.31; ES: 0.08, Table 1, Figure 3). In general, the load level showed no significant effect on the ROM (*p* = 0.541).

#### 3.3.2. Rotational Stiffness

The nail fixation showed significantly higher normalized RS values than the plate fixation for the mean of all force levels (*p* < 0.001; plate: 1.46 ± 0.33; nail: 1.80 ± 0.59; ES: 0.76, Table 1, Figure 3). The ES underlines this significance and presents a strong effect. The load level showed no significant impact on RS (*p* = 0.246).

#### 3.3.3. Diastasis

No significant difference was observed between the two methods and their influence on upper ankle diastasis for the mean of all force levels (*p* = 0.166; plate: 1.35 ± 0.26; nail: 1.46 ± 0.39; ES: 0.32, Table 1, Figure 3). ES showed a minor effect. The load level had no significant influence on the diastasis (*p* = 0.722).

## 4. Discussion

The purpose of this biomechanical cadaver study was to investigate whether the stabilization of unstable ankle fractures (PER injury stage 4 according to Lauge-Hansen [25]) using a Vitus-Fi Fibula Nail System is biomechanically comparable to locking plate fixation.

In our biomechanical study, both OS, ORIF using a locking plate, and CRNF using a fibula nail were observed to remain behind native stability in a highly unstable PER injury. A biomechanical comparison of the two OS showed comparable results. A significantly greater rotational stiffness was observed in the nail OS.

There is evidence in the clinical trials that the fibula nail may present advantages due to fewer wound complications [26,27,28]. A systematic review and meta-analysis conducted in 2022 that included randomized clinical trials concluded that there is good evidence for comparable clinical outcomes between the fibula nail and ORIF [29]. With moderate safety, to date, fewer postoperative complications can be expected [29]. A technique-related disadvantage is that closed reduction makes the anatomical restoration of the ankle difficult. These results suggest that nail fixation is a good alternative to open reduction and locking plate fixation for a geriatric patient population [13,18]. In addition, full weight-bearing should be possible postoperatively [18,22], which would offer clear advantages, especially for elderly people, because the absence of weight-bearing is often not possible at all.

In our biomechanical study, the comparison of the two techniques (nail and locking plate) with the corresponding native specimens showed significant differences in all three parameters (ROM, RS, and diastasis) toward reduced stability and rotational stiffness after fixation. It seems to be very clear that both fixation techniques failed to restore the native stability. With a PER (Stage 4) injury and a fracture segment at the Weber C level, a highly unstable situation was created in our study, which may be one explanation for the lower stability even after fixation. What this means for clinical recommendations is that it cannot be concluded from the biomechanical results alone. For functional stability under real-life conditions, the additional stabilizing effect of muscle forces and ankle joint passing tendons has to be considered. However, the question is how our results are reasonably put into practice, since we only tested specimens without muscles and soft tissues in the artificial injured area. We share an opinion similar to Switaj et al. on the question of weight-bearing following surgery. Due to the significant increase in diastasis following OS, weight-bearing should also be decided as a case decision depending on the severity of the injury [11]. When comparing the two fixation methods, there were no significant differences for the ROM and diastasis of the ankle. The effect sizes showed weak effects for ROM (ES = 0.08) and diastasis (ES = 0.32), indicating very comparable stability conditions with regard to these two parameters. However, there was a significant difference in RS to greater RS in nail fixation. The high ES value of 0.76 underlines the difference in the overall mean of the measured values. In contrast, the pairwise comparison showed no significant differences between specimens (Table 1). Overall, this result was unexpected regarding the existing literature. In contrast to Switaj et al., who observed a lower RS value for the nail than for the plate fixation across the syndesmosis, we could not demonstrate similar results. With regard to diastasis, Switaj et al. also observed no differences between nail and plate OS values. However, the nail probably remained below its potential in the study by Switaj et al. because only one syndesmotic screw was used [11]. This could be an explanation for the lower rotational stiffness observed by Switaj et al. [11]. This study also highlighted that diastasis showed even lower values for the locking plate compared to the native condition. The diastasis occurring following nail OS increased by only 0.7 mm. In our study, both OS values remained significantly lower than the native sample for all measurement parameters. Here, similar to Switaj et al.’s study, a dislocation model with a highly unstable situation was selected [11]. In addition to the complete transection of the syndesmotic ligaments and simulation of a fibular fragmentation zone, the deltoid ligament at the medial malleolus was also transected. This created a worst-case scenario, which has never before been biomechanically investigated for the fibula nail in this form and could be an explanation for the lower stability to the native condition. The study conducted by Smith et al. compared the fixation following a simulated supination-eversion injury (AO 44 B2, stage 3 according to Lauge-Hansen). Stability under an 800 N axial preload and external rotation loading was investigated [10]. The failure angle, the applied torque at failure, and the failure pattern were compared. In this case, the nail was only simply locked proximally and distally, and therefore most likely did not present the optimal results. In elderly patients, who frequently also present osteoporotic bone quality, the comparison of the lag screw and non-locking plate fixation did not seem optimal either. This was also illustrated by the nature of the failure observed in this study. In the non-locking one-third tubular plate construct, the screws failed, whereas in the nail, the lateral ligament structures ruptured. Thus, the nail seemed to be clearly superior to the non-locking plate construct. With a PER stage 4 injury, we created a more unstable situation than Smith et al. [10]. In addition, the comparison with a locking plate and dual syndesmotic screw provided a more clinically realistic comparison overall.

Carter et al. compared the fibula nail with a locking plate in a supination external rotation injury in geriatric specimens. However, the fibula nail was again used with only one syndesmotic screw [23]. The fixations were tested again to failure; therefore, a different biomechanical approach was employed than that used in our study. The comparison of OS using a nail with a syndesmotic screw versus a locking plate without stabilization of the syndesmosis does not seem to be a fair comparison. Despite the mean torque to failure result favoring the intramedullary nail by 1.9 Nm, the statistical significance was demonstrated only in the angle of failure, which favored the intramedullary nail by a mean of 13.2 degrees.

In our study, the fibula nail was tested for the first time, biomechanically fixed with two syndesmotic screws in addition to double distal locking, as required by the system. However, unlike the plate, these two screws were an integral part of the fibula nail system and inserted regardless of the stage of syndesmosis injury. To compare the fixation process, a standardized fracture model was fixed with two syndesmotic screws each for both the plate and nail. We observed an advantage here compared to the previous biomechanical studies, as the fibula nail obviously received an increase in stiffness and stability due to additional bony fixation points. From a biomechanical perspective, the fibula nail is shown to be an alternative to the locking plate.

### Limitations

A limitation of this biomechanical study was the low number of specimens, but this has similarly been reported in other comparable studies [22,23,24,25]. One explanation for the low number of specimens is certainly the availability of body donors, which is limited. The transfer of the biomechanical results to the clinic is not immediately possible due to the lack of muscular stabilization compared to living humans. Further clinical studies are needed to compare the two OS values in clinical practice. The instability model of PER injury reflects only a part of the possible injuries and instabilities at the ankle joint. An attempt was made to produce the worst-case scenario in order to maximally challenge the implants. Despite this, the results cannot simply be transferred to other injuries. Comparability to studies with failure testing is limited, as these were not performed. The biomechanical model chosen here with internal and external rotations did not correspond to the normal cyclic loading of an ankle joint in a walking cycle, but was an abstraction in the laboratory model.

## 5. Conclusions

The results show that both fixation techniques achieved comparable biomechanical stability values in the case of highly unstable ankle fractures. The nail offers an advantage over the plate for rotational stability. However, both fixations fell short of native stability and rotational stiffness. Therefore, full weight-bearing following osteosynthesis in a highly unstable situation such as pronation external rotation injury should not be considered in general terms, but rather on a case-by-case basis.

## Figures and Tables

**Table 1:** Results of the biomechanical tests of the nail and plate fixations for each load level, normalized to the native condition. Values greater than 1 indicate greater movement (range of motion (ROM) and diastasis) or stiffness; lower values vice versa. To compare both stabilization methods, the normalized values of ROM, RS, and diastasis were used in separate GLMs with the group as the fixed factor, respectively. The *p*-values and effect size (ES, Cohen’s d) are presented for each pairwise comparison and for the mean values of all force levels. The significance level was set at ≤0.05. The ES values as Cohen’s d mean: 0.2 = small effect, 0.5 = medium effect, 0.8 = large effect.

	Load Level, [Nm]	Nail	Plate	*p*-Value	ES
Normalized ROM [%]	2	0.90 ± 0.53	0.93 ± 0.40	0.896	0.07
	4	0.78 ± 0.35	0.81 ± 0.20	0.862	0.09
	6	0.72 ± 0.20	0.74 ± 0.13	0.854	0.10
	8	0.70 ± 0.17	0.72 ± 0.09	0.768	0.15
	10	0.72 ± 0.22	0.72 ± 0.07	0.974	0.02
	12	0.69 ± 0.12	0.72 ± 0.07	0.686	0.30
Mean values		0.75 ± 0.31	0.77 ± 0.22	0.694	0.08
Normalized RS [%]	2	1.59 ± 0.69	1.18 ± 0.35	0.172	0.73
	4	1.83 ± 0.59	1.40 ± 0.28	0.095	0.91
	6	1.91 ± 0.61	1.59 ± 0.40	0.253	0.60
	8	2.04 ± 0.62	1.59 ± 0.28	0.094	0.90
	10	1.78 ± 0.59	1.55 ± 0.29	0.416	0.50
	12	1.67 ± 0.23	1.45 ± 0.24	0.270	0.91
Mean values		1.80 ± 0.59	1.46 ± 0.33	<0.001	0.76
Normalized diastasis [%]	2	1.33 ± 0.47	1.20 ± 0.36	0.547	0.31
	4	1.43 ± 0.43	1.30 ± 0.33	0.527	0.33
	6	1.48 ± 0.39	1.40 ± 0.26	0.650	0.24
	8	1.51 ± 0.38	1.41 ± 0.17	0.512	0.33
	10	1.50 ± 0.47	1.39 ± 0.13	0.648	0.32
	12	1.49 ± 0.23	1.41 ± 0.16	0.582	0.41
Mean values	12	1.46 ± 0.39	1.35 ± 0.26	0.166	0.32

## Data Availability

The data presented in the study are stored on secure servers at University Hospital Jena. Donor consent forms are stored at the Anatomical Institute of the University Hospital in Jena. The data are available on request from the corresponding author.
